# Fabrication of a mercaptoacetic acid pillar[5]arene assembled nanochannel: a biomimetic gate for mercury poisoning†
†Electronic supplementary information (ESI) available. See DOI: 10.1039/c5sc04726a


**DOI:** 10.1039/c5sc04726a

**Published:** 2016-01-29

**Authors:** Fan Zhang, Junkai Ma, Yue Sun, Imene Boussouar, Demei Tian, Haibing Li, Lei Jiang

**Affiliations:** a Key Laboratory of Pesticide and Chemical Biology (CCNU) , Ministry of Education , College of Chemistry , Central China Normal University , Wuhan 430079 , P. R. China . Email: lhbing@mail.ccnu.edu.cn; b Beijing National Laboratory for Molecular Sciences (BNLMS) , Key Laboratory of Organic Solids , Institute of Chemistry , Chinese Academy of Sciences , Beijing , 100190 , P. R. China

## Abstract

Mercury ion binding blocks potassium ion channels, which leads to toxicity *in vivo*. It is challenging to design a simple and efficient artificial system to mimic the sophisticated biological process of mercury poisoning. Herein, based on biomimetic strategies, a tunable mercury(ii) ion-gate modulated by mercaptoacetic acid-pillar[5]arene (**MAP5**) is reported. By virtue of the unique design of the host–guest competition, potassium ion transport can actualize the reversible switching between “on” and “off” in the absence and presence of mercury ions. Moreover, the **MAP5**-immobilized nanochannel is highly effective at distinguishing Hg^2+^ from other metal ions and can be used to detect Hg^2+^ and act as an excellent and robust gate valve for developing integrated circuits and nanoelectronic logic devices. This study paves a new way for better understanding the physiological phenomenon of mercury toxicity and shows great promise for biomedical research.

## Abstract 2

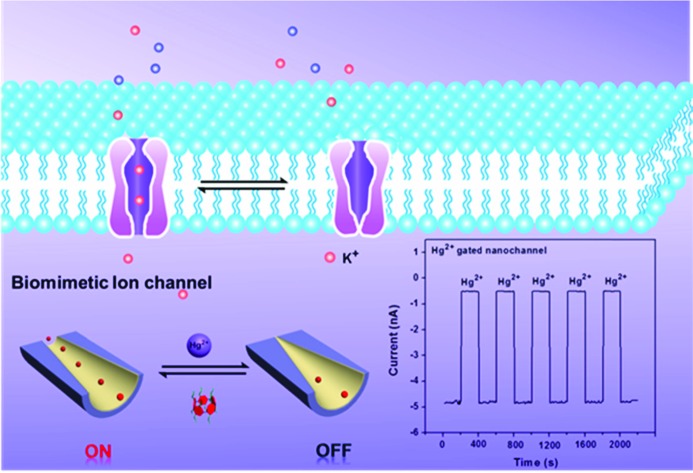
Herein, based on biomimetic strategies, a tunable mercury(ii) ion-gate modulated by mercaptoacetic acid-pillar[5]arene (**MAP5**) is reported.

## Introduction

The mercury(ii) ion is a highly toxic pollutant that can cause serious damage to nervous tissues and organs, such as lung damage, deterioration of the brain and kidneys, and so forth.^1^–^6^ Recently, a number of studies have demonstrated that mercury ion binding blocks potassium ion channels, which leads to toxicity *in vivo*.^7^–^9^ The biological process is complicated, which is quite significant when investigating the pathology and toxicology. Therefore, it is hugely challenging to fabricate a simple and efficient artificial device to mimic the biological process of mercury(ii) ion gated potassium ion channels.

The gating of biological ion channels is the basis of cellular signal-transduction processes.^10^,^11^ It always plays a crucial role in regulating the functionalities of biological channels, such as in opening and closing the ion channel, governing specific ion diffusion, and controlling ion conduction in response to specific stimuli.^12^–^15^ However, most of the biological channels in nature, embedded in lipid bilayers, are not stable and sophisticated.^16^,^17^ To better understand the complicated process of biological ion transport, artificial biomimetic nanochannels have been widely developed because of their excellent mechanical robustness and chemical stability.^18^–^22^ These solid-state synthetic nanochannels possess great flexibility in terms of their geometry and size, and have multi-functional surface properties.^23^–^26^ Especially the conical nanochannel in polyethylene terephthalate (PET), which has strong implications for the simulation of the different ionic transport processes as well as the enhancement of the functionality of biological ion channels.^27^–^30^ Recently, interest in nanochannels has been stimulated by discoveries of the importance of biological channels in many of the physiological processes of living organisms as well as in building functional gates. Hence, preparing nanochannels as smart switchable gates to mimic mercury ions poisoning has drawn enormous research attention.

**Scheme 1:**
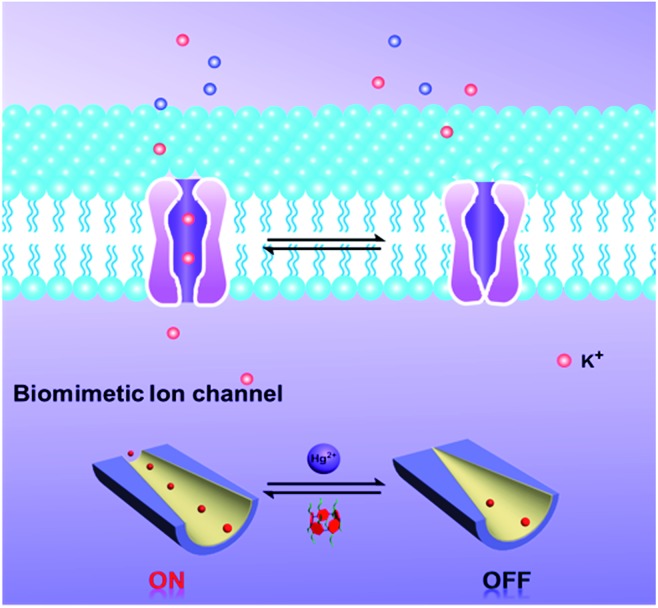
Schematic diagram of the design of a biomimetic mercury ion-gated nanochannel.

To the best of our knowledge, among the various methods for constructing switchable gates, host–guest binding and release can be used as a simple and robust method to fabricate “on–off” switches. Pillar[5]arenes, as a new type of macrocyclic host, have gained increasing attention in recent years.^31^–^33^ They have been used extensively as supramolecular switches for host–guest interactions, such as with pseudorotaxanes, rotaxanes, catenanes, supramolecular dimers, and so forth.^34^–^36^ With these in mind, we developed a new strategy to introduce molecular switches into nanochannel systems to fabricate a tunable mercury ion-gated nanochannel. Herein, inspired by the phenomenon of mercury ions binding with thiol containing protein blocks in the potassium ion channels, we designed and synthesized a water-soluble mercaptoacetic acid-pillar[5]arene (**MAP5**) using the “thiol–ene” click reaction. By virtue of host–guest interactions, **MAP5** can assemble into the inner wall of the nanochannel which is modified with suitable guest molecules. Because mercury ions bind to thiol-containing molecules, mercury ions can remove **MAP5** from the host–guest complex and change the surface charge and wettability of the nanochannel. Such a tunable mercury(ii) ion gate with good molecular responsive properties can open and close in response to external stimuli and control potassium ion transport in the channels (Scheme 1).

## Results and discussion

### Synthesis of mercaptoacetic acid-pillar[5]arene

**Fig. 1:**
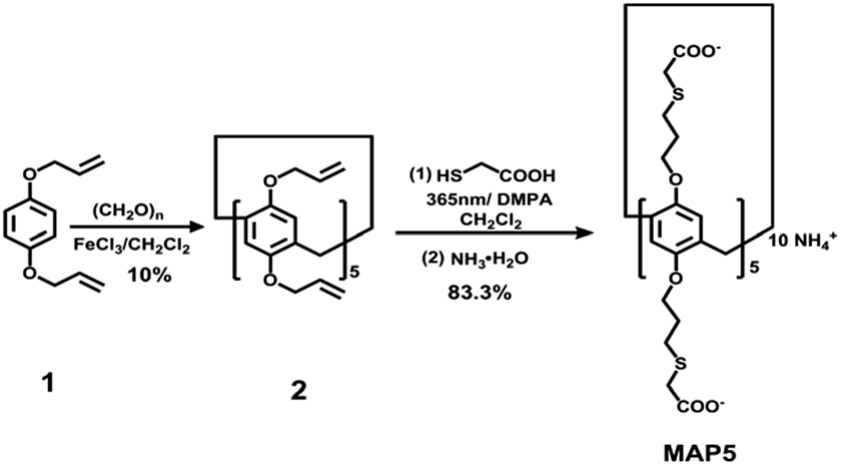
Design and highly efficient synthesis of water-soluble mercaptoacetic acid-pillar[5]arene.

To obtain a Hg^2+^ responsive supramolecular switch that can be assembled in water, we designed and synthesized water-soluble mercaptoacetic acid-pillar[5]arene (**MAP5**) using a rapid photocatalytic click reaction. The strategy for **MAP5** synthesis is shown in Fig. 1. Paraformaldehyde (0.9 g, 30 mmol) was added to a solution of monomer **1** (1.9 g, 10 mmol) in dichloromethane (30 mL) under a nitrogen atmosphere. Iron(iii) chloride (FeCl_3_, 0.325 g, 2 mmol) was then added to the solution and the mixture was stirred at room temperature for 30 min. After the solvent was removed, the obtained solid was purified using column chromatography on silica gel with petroleum ether/ethyl acetate (40 : 1 v/v) as the eluent to obtain white powder **2** (0.2 g, 10% yield). Subsequently, **MAP5** was synthesized using the classical “thiol–ene” click reaction. Mercaptoacetic acid (0.736 g, 8 mmol) and the photoinitiator 2,2-dimethoxy-2-phenylacetophenone (DMPA, 50 mg) were added to compound **2** (0.2 g, 0.2 mmol) in dichloromethane (20 mL) and exposed to 365 nm UV light under stirring at room temperature for 15 min. After solvent evaporation, the crude product was purified using column chromatography to give a white powder. Then the product was mixed with 40% NH_3_·H_2_O (10.0 mL) and stirred at reflux for 5 h. The mixture was concentrated under reduced pressure to obtain the precipitated product. The product was collected by filtration, washed with ethanol, and dried under vacuum to obtain **MAP5** as a white solid (0.214 g, 83.3% yield), which was characterized using ^1^H NMR and ^13^C NMR spectroscopy, and MALDI-TOF (see Fig. S6–S8†).

### The ^1^H NMR analysis of the supramolecular switch

**Fig. 2:**
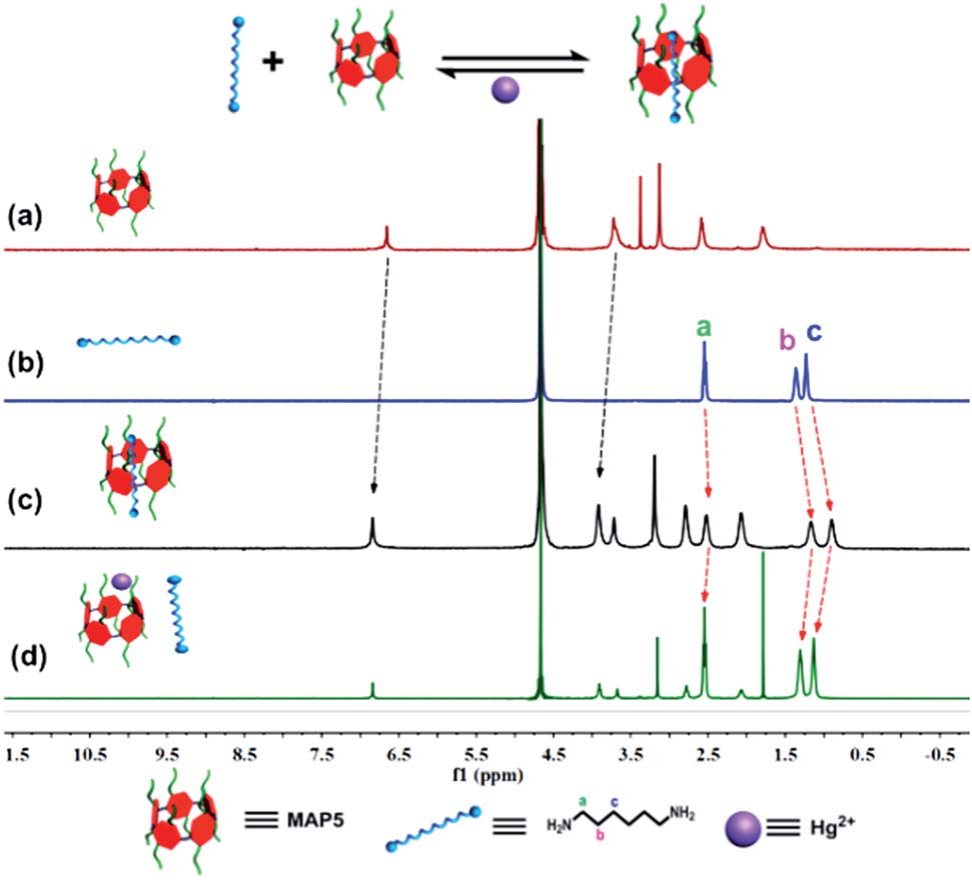
^1^H NMR spectra (D_2_O, 400 MHz, 298 K) of (a) **MAP5** (b) HDA (c) **MAP5** + HDA and (d) the addition of mercury acetate (Hg^2+^) to the **MAP5**–HDA system. The spectra show that Hg^2+^ has a stronger affinity for **MAP5** than HDA and can result in HDA being released from the **MAP5**–HDA complex.

Based on molecular recognition, we devised a new strategy to introduce pillar[5]arene to the nanochannel using host–guest interactions. We chose 1,6-hexanediamine (HDA) as a model guest molecule immobilized on the surface. Initially, ^1^H NMR spectroscopy was performed to investigate the host–guest interaction of **MAP5** and HDA. As shown in Fig. 2, when 1.0 equiv. of HDA was added to a solution of **MAP5**, chemical shift changes of some protons in HDA and **MAP5** appeared (Fig. S9†). The protons H_a_, H_b_ and H_c_ of the HDA alkyl chain exhibited substantial upfield shifts of Δ*δ*_a_ = 0.062 ppm, Δ*δ*_b_ = 0.164 ppm, and Δ*δ*_c_ = 0.23 ppm, respectively, because of the inclusion-induced shielding effects when interacting with **MAP5**. Simultaneously, the proton of benzene in **MAP5** was also downshifted by Δ*δ* = 0.206 ppm. These shifts can be attributed to the alkyl chain being inserted into the cavity of **MAP5** to form a host–guest complex. Details of the interaction of HDA and **MAP5** were measured through ^1^H NMR titration. The mole ratio plots based on the proton NMR data showed that the complexes of **MAP5** and HDA had a 1 : 1 stoichiometry in water at room temperature (as shown in Fig. S10 and S11†). Moreover, computational calculations at the b3lyp/6-31G (d) levels verified the formation of a host–guest complex driven by hydrophobic interactions (Fig. S12†). When mercury(ii) ions were introduced into the **MAP5**–HDA system, the chemical shift changes of HDA recovered because of the stronger affinity between Hg^2+^ and **MAP5**. On the other hand, the protons in **MAP5** also exhibited slight chemical shift changes due to the interactions with Hg^2+^. These results revealed that Hg^2+^ successfully competes with HDA to form the **MAP5**–Hg^2+^ complex.

### Construction of the supramolecular switch on the PET planar film

Encouraged by the above competition between Hg^2+^ and HDA in aqueous solution, we further investigated the tunable switch from the functionalized poly(ethylene terephthalate) (PET) membrane surface properties. In this experiment, to confirm that HDA and **MAP5** modified the surface of the PET film successfully, the functionalized film was characterized using contact angle (CA) measurements and X-ray photoelectron spectroscopy (XPS) analysis. Fig. S14† shows that the CA of the etched membrane with its exposed –COO^–^ groups is 61.9° ± 2.3°. After modification with HDA, the functionalized surface has a CA of 76.4° ± 0.2°. Then, when **MAP5** attached to the HDA functionalized membrane by self-assembly, the membrane became more hydrophilic due to the multiple –COO^–^ groups of **MAP5** and had a CA of 54.7° ± 0.6°. Furthermore, XPS was used to evaluate the chemical composition of the PET membrane. Before modification (black line), the spectrum showed only a C 1s peak at 284.83 eV and an O 1s peak at 531.89 eV. Then the N 1s peak at 399.62 eV appears in the modified membrane owing to the nitrogen atom in HDA. The S 2p peak appears at 163.43 eV after **MAP5** self-assembles on the surface of the PET membrane. Normally, the density of –COO^–^ groups on the surface was estimated to be approximately 1 group per nm^2^.^37^ Therefore, according to the XPS derived relative content of N and S, we calculated the density of **MAP5** modified on the surface to be approximately 0.01 **MAP5** molecules per nm^2^ (see Fig. S15 and Tables S2–S4†).

**Fig. 3:**
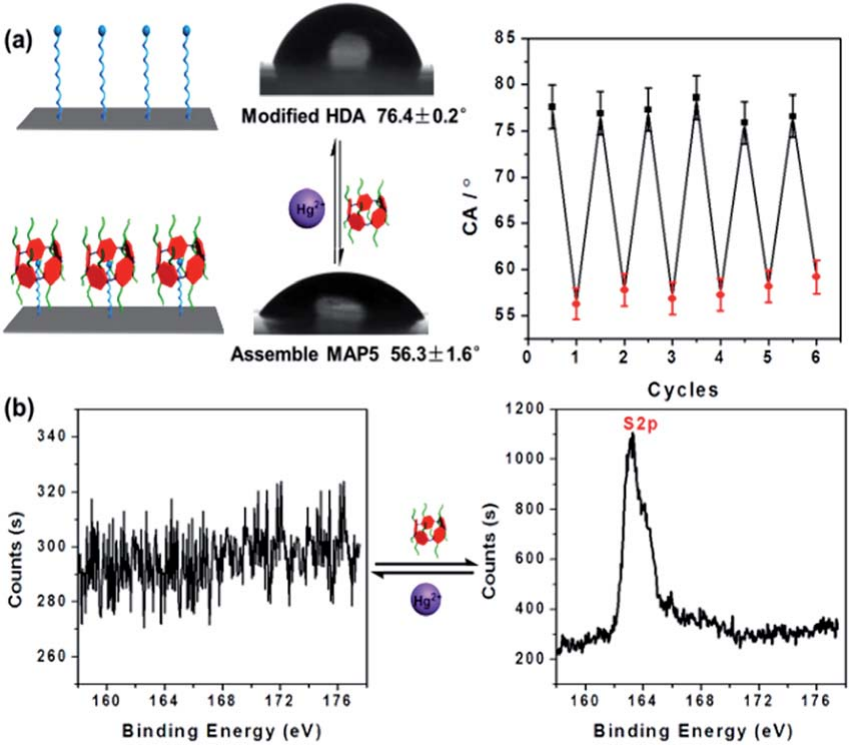
Reversible properties of the molecular switch on the PET planar film by (a) CA and (b) XPS characterization. It shows that the molecular switch can be well regulated by Hg^2+^ and **MAP5**.

As wettability plays a crucial role in the switchable system of HDA–**MAP5**–Hg^2+^, we further investigated the wettability of the molecular switch using a HDA-modified PET planar film (Fig. 3a). The results show that before **MAP5** assembled on the surface of the film, it appears to be hydrophilic with a CA of 76.4° ± 0.2°. The film surface containing **MAP5**, through the host–guest interaction with HDA, shows a significantly hydrophilic surface with a CA of 56.3° ± 1.6°. Upon addition of Hg^2+^, the CA returned to that of the HDA-modified film, which indicates that Hg^2+^ can remove **MAP5** from the complex of HDA–**MAP5** because of the high affinity between Hg^2+^ and **MAP5**. Moreover, the CA of the HDA film alternately immersed in **MAP5** aqueous solution and Hg^2+^ aqueous solution could reversibly switch between 76.4 ± 0.2° and 56.3 ± 1.6°. Hence, according to these results, a cycling experiment of the HDA-modified PET surface in **MAP5** and Hg^2+^ aqueous solutions was carried out. The CA switched six times, indicating the good reversible change of the wettability of the surface as a molecular switch. In addition, application of the HDA-modified PET surface as a molecular switch was also confirmed using XPS characterization. As shown in Fig. 3b, the S 2p peak at 163.43 eV is not present for the HDA-modified film, but when the HDA immobilized surface assembles with **MAP5**, the peak is clearly observed. After immersing the film in 1 mM Hg^2+^ solution and washing with deionized water, the S 2p peak disappears, which is caused by **MAP5** being removed from the HDA film due to its stronger affinity for Hg^2+^. These results clearly demonstrate the excellent reversible properties of the HDA–**MAP5** complex toward Hg^2+^.

### Fabrication of the **MAP5** assembled nanochannel

**Fig. 4:**
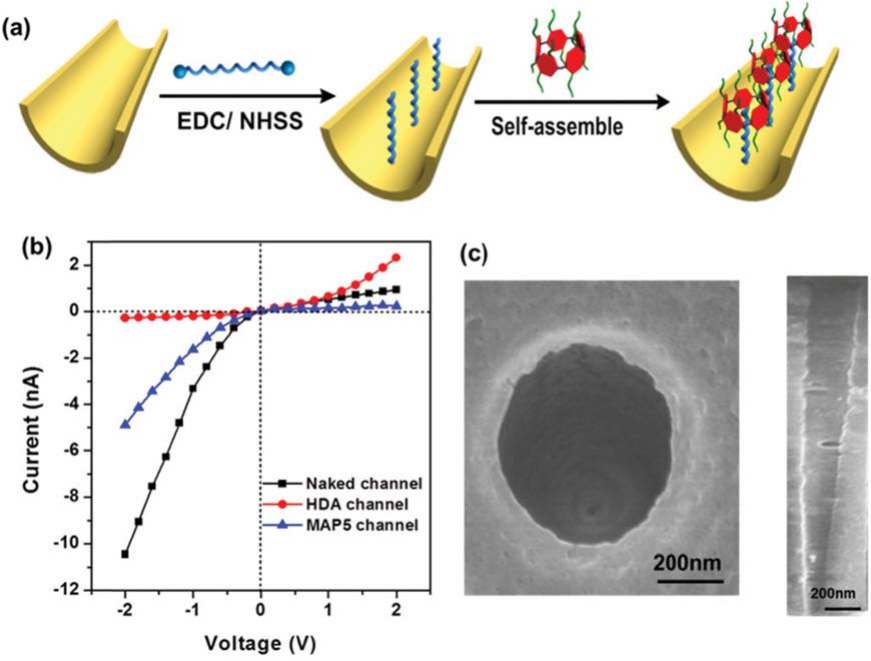
(a) Schematic description of the modification process in the nanochannel. (b) *I*–*V* characteristics of **MAP5**-assembled nanochannel. (c) The top scan of a typical conical PET nanochannel observed using SEM. The result shows that the nanochannel was successfully fabricated.

Based on the above properties in solution and on the PET surface, we attempted to fabricate a tunable mercury ion-gated nanochannel with the idea of the above supramolecular switch. Firstly, a single conical nanochannel was prepared using an asymmetric track-etch technique on a 12 μm thick PET membrane (Hostaphan RN12 Hoechst) with a single heavy-ion-irradiated track in the center. Before the etching process, each side of the PET membrane was exposed to UV light (365 nm) for 1 hour. Diameter measurements of the nanochannels were performed with a commonly used electrochemical method.^38^ The large opening (base) was about 560 nm in diameter and the tip diameter was calculated to be around 20 nm (see Fig. 4c and S17†). During the chemical etching process, carboxyl groups (–COO^–^) were exposed on the nanochannel surface, and then 1,6-diaminohexane (HDA) covalently linked to the –COO^–^ groups by a classical EDC/NHSS cross-linking reaction. With the host–guest interaction, we fabricated the mercury(ii) ion-gated system by adding a solution of **MAP5** (1 mM) to the HDA-immobilized channel for self-assembly. After a further 1 hour, we removed the solution and washed the membrane with pure water three times. Ion current measurements were carried out using a Keithley 6487 picoammeter (Keithley Instruments, Cleveland, OH) in a custom-designed electrolyte cell, and the sample membrane was mounted between the two halves of the cell (see Fig. S16†). All of the experiments were carried out at room temperature (25 °C).

The ion transport properties of the single nanochannel before and after **MAP5** assembly were investigated by measuring current–voltage (*I*–*V*) curves with 0.1 M KCl solution as an electrolyte at a neutral pH value (pH 6.86) on both sides of the membrane. As shown in Fig. 4b, the original nanochannel showed a nonlinear *I*–*V* curve at neutral pH because of the presence of inherent anionic carboxylate groups (–COO^–^), which can preferentially transport cations (K^+^) from the tip entrance to the base side of the channel when a potential is applied across the membrane. After the chemical modification with HDA, there is an obvious decrease in the ionic current at –2 V and the immediate inversion of the rectifying characteristics indicate that successful modification by HDA resulted in a change in the polarity of the nanochannel from negative to positive (red line in Fig. 4b). Subsequently, **MAP5** was attached to the nanochannel through self-assembly with HDA by the host–guest interaction. The rectifying characteristic returned to the initial state of cation (K^+^) selectivity due to the rich negative charges coming from **MAP5**.

### The tunable properties of the mercury ion gated nanochannel

**Fig. 5:**
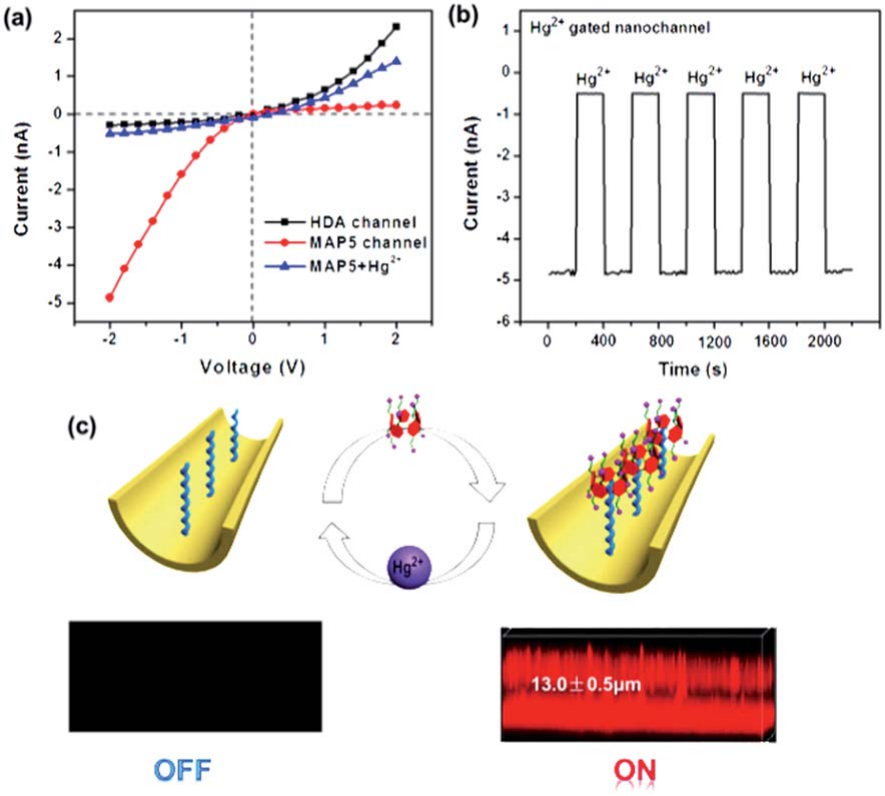
(a) *I*–*V* characteristics of the switchable nanochannel modulated by **MAP5** and Hg^2+^. (b) Stability and responsive switching ability of the tunable system. Reversible variation of potassium ion current was measured at a negative potential (–2 V) with the alternate addition of Hg^2+^ and **MAP5**. (c) Laser scanning confocal microscopy observation of the rhodamine B derivative fluorescent pillar[5]arene assembled on the HDA-modified nanochannel surface and competition with Hg^2+^. The “on–off” fluorescence signal indicates that **MAP5** can assemble on the nanochannel and is removed by Hg^2+^.

The tunable switch of this ion-gated nanochannel was evaluated by testing the current change with the addition of Hg^2+^ and **MAP5**. As shown in Fig. 5a, in the presence of **MAP5**, the surface of the nanochannel is hydrophilic and negatively charged, which leads to a high ion current and low resistance. Under these conditions, the gate shows the “on” state for potassium ion selective transport, and the current through the **MAP5** assembled nanochannel is about –4.8 nA at a voltage of –2 V. After immersing the film in a solution of 1 mM Hg^2+^, the current at –2 V remarkably decreases to –0.5 nA because **MAP5** is removed from the **MAP5**–HDA complexes. The pseudorotaxane complex transforms to the positively charged HDA-modified state, and the gate shows the “off” state for potassium ion transport inhibition. The HDA-modified nanochannel then recovers the function for binding **MAP5**. The reversibility of the ion current measured with the addition of **MAP5** and Hg^2+^ could be repeated for six cycles. After several cycles, there was only a slight decrease in the ion current, indicating that the nanochannel is stable. Hence, the measurements confirm that this tunable system can be switched between the “on” and “off” states in response to **MAP5** and Hg^2+^, and it can regulate potassium ion transport in the presence of Hg^2+^.

To further demonstrate the reversibility of the nanochannel, laser scanning confocal microscopy was conducted. We used the fluorescent **MAP5** derivative (**MAP5**–RhB), which was synthesized by linking the carboxyl group (–COO^–^) of **MAP5** to rhodamine B amine (RhB–NH_2_). A host–guest complex was then formed on the HDA-modified porous PET membrane by the interaction between HDA and **MAP5**–RhB. As shown in Fig. 5c, when **MAP5**–RhB successfully assembles on the HDA immobilized nanochannel, a fluorescence signal appears (the “on” state). The fluorescence thickness is *ca.* 13.0 ± 0.5 μm, which agrees with the actual thickness of the PET membrane. When Hg^2+^ is added to the system, the fluorescence is significantly quenched (the “off” state) as the **MAP5**–RhB is removed from the nanochannel. Clearly, all the above data show that the **MAP5** assembled nanochannel exhibits an excellent Hg^2+^ recognition capability and can act as an excellent model to mimic sophisticated physiological processes.

### The ion selectivity of the **MAP5** assembled nanochannel

**Fig. 6:**
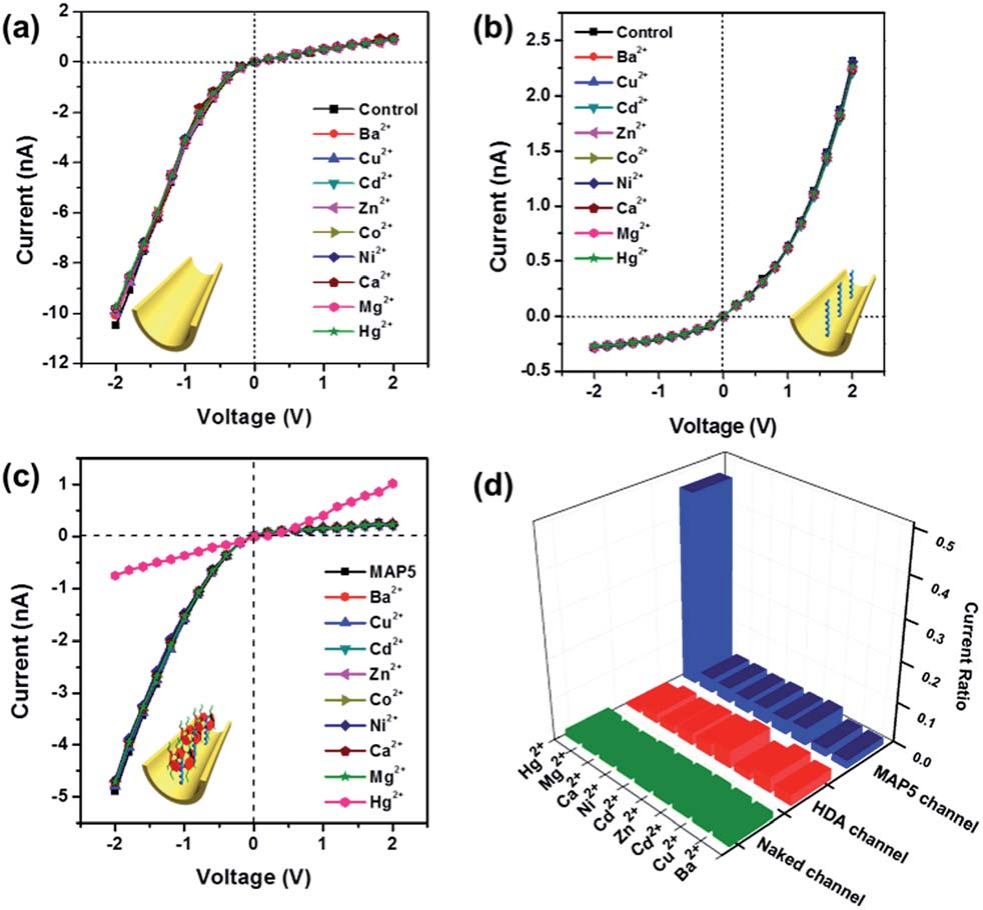
*I*–*V* curves of the nanochannel in the presence of 10 μM Ba^2+^, Cu^2+^, Cd^2+^, Zn^2+^, Co^2+^, Ni^2+^, Ca^2+^, Mg^2+^, and Hg^2+^. (a) The naked channel. (b) The HDA immobilized nanochannel. (c) **MAP5** assembled nanochannel. (d) Histogram of ionic current change ratios ((*I* – *I*_0_)/*I*_0_) at –2 V after adding the above ions into the nanochannels. The results show that little change was observed for the naked nanochannel and HDA immobilized nanochannel.

Additionally, the **MAP5** assembled nanochannel could also have good Hg^2+^ selective gated properties. Fig. 6 shows *I*–*V* curves of the **MAP5** assembled nanochannel exposed to electrolytes containing 10 μM Ba^2+^, Cu^2+^, Cd^2+^, Zn^2+^, Co^2+^, Ni^2+^, Ca^2+^, Mg^2+^, and Hg^2+^. The presence of Hg^2+^ results in a drastic decrease in the ion flux across the **MAP5**-assembled nanochannel, whereas the current is nearly constant in the presence of the other tested ions. This can be ascribed to the thiol of **MAP5** having a stronger affinity for Hg^2+^ than the other ions. Thus, other metal ions cannot change the surface properties of the nanochannel. The current-change ratio ((*I* – *I*_0_)/*I*_0_) at –2 V was determined to quantify the changes in the ion current passing through the modified nanochannels in the presence of different metal ions. Compared with the current ratios from the naked channel and HDA channel, we found that only the **MAP5** assembled channel can act as a good selectivity binding site for Hg^2+^. Moreover, the Hg^2+^-responsive properties were also confirmed using UV-vis spectroscopy (Fig. S13†), from which we found that only Hg^2+^ can enhance the characteristic peak. Therefore, these results indicate that Hg^2+^ can efficiently control the “off” and “on” states in the **MAP5**-modified nanochannel.

To further illustrate the gating ratio of the mercury ion regulated nanochannel, the current–concentration (*I*–*C*) properties of the **MAP5**-assembled nanochannel for Hg^2+^ were determined. As shown in Fig. S18,† the transmembrane ion current at –2 V dramatically decreases with increasing Hg^2+^ concentration. With a Hg^2+^ concentration range from 1 nM to 1 μM (Fig. S18a†), the current gradually decreases, which indicates that the gate is closed. Therefore, the gating ratio can be defined as the current changing ratio. From the results, with 1 nM Hg^2+^ in the system, the potassium current is close to 6.5%. Moreover, when the concentration of Hg^2+^ is more than 1 μM, the current sharply decreases and there is gradual inversion of the *I*–*V* curve. This illustrates that Hg^2+^ can bind with **MAP5** and form a stable **MAP5**–Hg^2+^ complex, gradually removing **MAP5** from the HDA-modified nanochannel, which can act as a good gate for regulating and controlling potassium ion transport.

## Conclusions

In summary, a biomimetic tunable mercury(ii)-gated nanochannel was successfully developed by immobilizing **MAP5** on a HDA-functionalized nanochannel. The system shows a responsive switching ability and stability by virtue of the unique host–guest interaction. This bioinspired ion gate systematically regulates potassium ion transport in the presence of mercury ions. When the channel shows the “on” state, potassium ion transport proceeds freely, representative of a normal biological organism; when it shows the “off” state, the potassium ion channel is blocked, indicating mercury poisoning. This work provides an interesting insight to comprehensively understand the important physiological process of mercury(ii) ions binding with and blocking potassium ion channels, which results in toxicity. Moreover, the device easily distinguishes Hg^2+^ from other metal ions with a detection limit of 1 nM, which has potential applications in biosensors, toxicological testing for Hg^2+^, and as an excellent and robust gate valve for developing integrated circuits and programmable nanoelectronic logic devices.

## Experimental section

### Nanochannel preparation

The single conical nanochannel was produced in a PET membrane (Hostaphan RN12 Hoechst, 12 μm thick, with single ion tracks in the center) using an asymmetric track-etched technique. Briefly, the PET membrane was embedded between the two chambers of a conductivity cell at about 35 °C. One chamber was filled with etching solution (9 M NaOH), while the other side was filled with stopping solution (1 M HCOOH + 1 M KCl) (Fig. S16†). For observation of the etching process, the voltage (1 V) used to monitor the etching process was applied in such a way that the transmembrane ion current could be observed as soon as the nanochannel opened. The etching process was stopped at a desired current value corresponding to a certain tip diameter. Then the membrane was soaked in MilliQ water (18.2 MΩ) to remove residual salts.

### Modification

As a result of the chemical etching, carboxyl groups are generated on the nanochannel surface. These can be activated with EDC/NHSS, forming an amine-reactive ester intermediate. Then these reactive esters were further condensed with 1,6-hexanediamine (HDA) through the formation of covalent bonds. In this paper NHSS ester was formed by soaking PET film in an aqueous solution of 15 mg of EDC and 3 mg of NHSS for 1 hour. After that the film was washed with distilled water and treated with 1 mM HDA solution overnight. Finally, the modified film was washed three times with distilled water. Then, mercaptoacetic acid-pillar[5]arene (**MAP5**) was attached to the HDA-channel through self-assembly.

### Ion current measurements

Ion currents were measured using a Keithley 6487 picoammeter (Keithley Instruments, Cleveland, OH). Ag/AgCl electrodes were used to apply a transmembrane potential across the film. The film was mounted between the two halves of a conductance cell. Both halves of the cell were filled with 0.1 M KCl, pH 6.86. In order to record the *I*–*V* curves, a scanning triangle voltage signal from –2 V to +2 V with a 40 s period was selected. Each test was repeated 5 times to obtain the average current value at different voltages.

### XPS

X-ray photoelectron spectra (XPS) data were obtained with an ESCALab220i-XL electron spectrometer from VG Scientific using 300 W Al Kα radiation. All peaks were referenced to C 1s (CHx) at 284.8 eV in the deconvoluted high resolution C 1s spectra.

### Contact angle measurements

Contact angles were measured using an OCA 20 contact angle system (Dataphysics, Germany) at 25 °C. Before the contact angle test, the sample was blown dry with N_2_. In each measurement, a droplet of about 2 μL of water was dispensed onto the surface of the PET membrane. The average contact angle value was obtained at five different positions of the same membrane.

### Confocal fluorescence images

Confocal images were acquired using a Zeiss confocal laser scanning unit mounted on an LSM710 fixed-stage upright microscope.

### Gaussian calculation

Computational calculations were carried out at the density functional theory b3lyp/6-31G (d) levels using Gaussian 03.

## Supplementary Material

Supplementary information
