# Supplementary material for: Fabrication of a mercaptoacetic acid pillar[5]arene assembled nanochannel: a biomimetic gate for mercury poisoning
Source: Chem Sci. 2016 Jan 29;7(5):3227–33. doi: 10.1039/c5sc04726a (PMC6005340; doi:10.1039/c5sc04726a)
Supplement: Supplementary file 1 [file SC-007-C5SC04726A-s001.pdf]

Electronic Supplementary Material (ESI) for Chemical Science.
This journal is © The Royal Society of Chemistry 2016

Supporting Information

Fabrication of Mercaptoacetic acid Pillar[5]arene Assembled Nanochannel: A Biomimetic Gate for Mercury Poisoning

Fan Zhang, † Junkai Ma, † Yue Sun, † Imene Boussouar, † Demei Tian, † Haibing Li*†‡ and Lei Jiang‡

† Key Laboratory of Pesticide and Chemical Biology (CCNU), Ministry of Education, College of Chemistry, Central China Normal University, Wuhan 430079, P. R. China

‡ Beijing National Laboratory for Molecular Sciences (BNLMS), Key Laboratory of Organic Solids, Institute of Chemistry, Chinese Academy of Sciences, Beijing 100190, P. R. China.

Email: lhbing@mail.ccnu.edu.cn

Contents of Supporting Information:

1. Materials ……………………………………………………………………………………S2

2. Synthetic procedures for mercaptoacetic acid -pillar[5]arene ………………………………...S3-S5

3. ^{1}H NMR, ^{13}C NMR and MS for the main compounds ……………………….......................S6-S13

4. ^{1}H NMR of the host-guest interaction ……………………………………………………...S14-S16

5. Gaussian calculation for the interaction of MAP5 and HDA………………………………………S17

6. UV-vis characteristic for the selective of MAP5 to metal ions ………………………...............S18

7. Contact angles measurement …………………………………………………………………S19

8. XPS Characterization ………………………………………………………………………S20-S21

9. Nanochannel fabrication ……………………………………………………………………..S22

10. SEM Characterization ………………………………………………………………………S23

11. Modification process ………………………………………………………………………..S24

12. Ion currents measurement …………………………………………………………………..S25

13. Laser scanning confocal microscopy………………………………………………………...…S26

14. References………………………………………………………………………………...S27

1. Materials

Poly (ethylene terephthalate) (PET, 12 μm thick) membranes were irradiated with single heavy ion (Au) of energy 11.4 MeV/nucleon at UNILAC linear accelerator (GSI, Darmatadt, Germany). 1-Ethyl-3-(3-dimethyllaminopropyl) carbodiimide hydrochloride (EDC • HCl, ⩾98.5%), N-hydroxysulfosuccinimide (NHSS, ⩾98.0%), 1,6-hexanediamine (HDA), sodium hydroxide (NaOH), hydrochloric acid (HCl), formic acid (HCOOH), potassium chloride (KCl) were purchased from Sinopharm Chemical Reagent Shanghai Co., Ltd. (SCRC, China). Mercaptoacetic acid -pillar[5]arene was synthesized by the steps below.(Scheme S1) All chemical reagents were all used as received, electrolyte solution were prepared in MilliQ water (18.2 MΩ). Current-voltage curves were measured by a Keithley 6487 picoammeter (Keithley Instruments, Cleveland, OH). Confocal fluorescent images were acquired using a Zeiss confocal laser scanning unit mounted on a LSM710 fixed-stage upright microscope. UV-vis absorption spectra were recorded on UV-2501.

2. ***Synthetic procedures for mercaptoacetic acid -pillar[5]arene.***

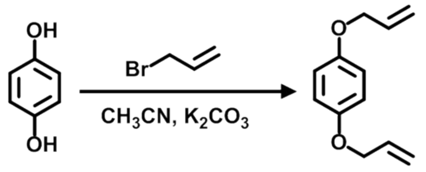

1

Scheme S1. Synthesis of compound **1**.

As shown in Scheme S1, compound **1** was synthesized according to the literature [S1]. Primarily, potassium carbonate (K_2CO_3, 17.25 g, 125 mmol) was added to a solution of *p*-dihydroxybenzene (5.5 g, 50 mmol) in acetonitrile (100 mL) under nitrogen atmosphere. Then bromopropylene (15.12 g, 10.6 mL, 125 mmol) was added to the solution and the mixture was reflux overnight. After the mixture was cooled, the solid was removed by filtrated, the solution dried under vacuum and purified by column chromatography on silica gel with petroleum ether/ ethyl acetate (20:1 v/v) as the eluent to get a colorless tabular crystal **1** (7.5 g, yield 79%). ^{1}H NMR ($CDCl_3$, 400 MHz, ppm) δ 6.84 (m, 4H, Ar-H), 6.08-6.01 (m, 2H, CH=CH_2), 5.41-5.37 (m, 2H, CH=CH_2), 5.28-5.25 (m, 2H, CH=CH_2), 4.47 (d, 4H, OCH_2, J = 5.2 *Hz*). ^{13}C NMR (100 MHz, $CDCl_3$, 298 K) δ 152.79, 133.52, 117.37, 115.54, 69.33. The result is consistent with the literature report [S1].

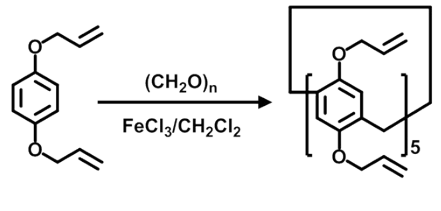

2

Scheme S2. Synthesis of compound **2**.

Compound **2** was synthesized according to the literature S2. To a solution of monomer **1** (1.9 g, 10 mmol) in dichloromethane (30 mL) was added paraformaldehyde (0.9 g, 30 mmol) under nitrogen atmosphere. Then Iron (III) chloride ($FeCl_3$, 0.325 g, 2 mmol) was added to the solution and the mixture was stirred at room temperature for 30min. After the solvent was removed, the obtained solid was purified by column chromatography on silica gel with petroleum ether/dichloromethane (40:1 v/v) as the eluent to get a white powder **2** (0.2 g, yield 10%). ^{1}H NMR ($CDCl_3$, 400 MHz, ppm) δ 6.75 (s, 10H, Ar-H), 5.89-5.82 (m, 10H, CH=CH_2), 5.23 (d, J = 17.1 Hz, 10H, CH=CH_2), 5.02 (d, J = 10.4 Hz, 10H, CH=CH_2), 4.30 (d, J = 5.1 Hz, 20H, OCH_2), 3.79 (s, 10H, Ar-CH_2). ^{13}C NMR (100 MHz, $CDCl_3$) δ (ppm): 149.30, 133.90, 128.40, 116.71, 115.01, 69.03, 29.63. MS (ESI) calcd for m/z: 1010.5; found: 1011.503 $[M+H]^+$. Anal. calcd for $C_{65}H_{70}O_{10}$: C,77.20; H, 6.98; found: C, 77.23; H, 6.94.

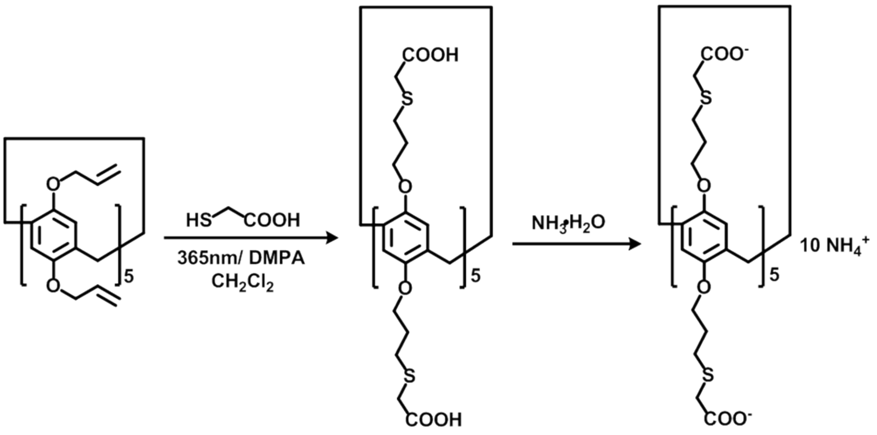

3 4

Scheme S3. Synthesis of mercaptoacetic acid pillar[5]arene.

Water-soluble mercaptoacetic acid-pillar[5]arene was synthesized in the steps. Compound **2** (0.2 g, 0.2 mmol) in dichloromethane (20 mL) added in thioglycollic acid (0.736 g, 8 mmol), and then a photoinitiator [2, 2-dimethoxy-2-phenylacetophenone (DMPA)] (50 mg) as catalyst was added with 365 nm UV stirred in dichloromethane at room temperature for 15 min. After solvent evaporation, the crude product was purified by column chromatography to give **3** as a white powder (0.32g, yield 85%). Then, the water-soluble mercaptoacetic acid-pillar[5]arene (MAP5) synthesized by a solution of **3** (0.20 g, 0.104 mmol) and 40% $NH_3•H_2O$ (10.0 mL) stirred at reflux for 5 h. The mixture was concentrated under reduced pressure to get the precipitated product. Then it was collected by filtration, washed with ethanol and dried under vacuum to obtain MAP5 as a white solid (0.214 g, 98%). ^{1}H NMR (D_2O, 400 MHz, ppm) δ 6.66 (s,10H, Ar-H), 3.72(s, 20H, S-CH_2-COOH), 3.37 (s, 10H, ArCH_2Ar), 3.13(m, 20H, ArOCH_2), 2.58(m, 20H, S-CH_2), 1.79(m, 20H, CH_2). ^{13}C NMR (DMSO-*d6*, 100 MHz, 298 K): δ 172.08, 149.02, 127.82, 114.12, 66.07, 33.92, 30.66, 28.93, 28.57. MALDI-TOF-MS calcd for m/z: $[M-10NH_3+Na]^+$ 1953.7, found: 1953.676. Anal. Calcd for $C_{85}H_{140}N_{10}O_{30}S_{10}$: C, 48.55; H, 6.71; N, 6.66; S, 15.25; found: C, 48.58; H, 6.82; N, 6.54; S, 15.41.

3. *^{1}H NMR, ^{13}C NMR and MS for the main compounds.*

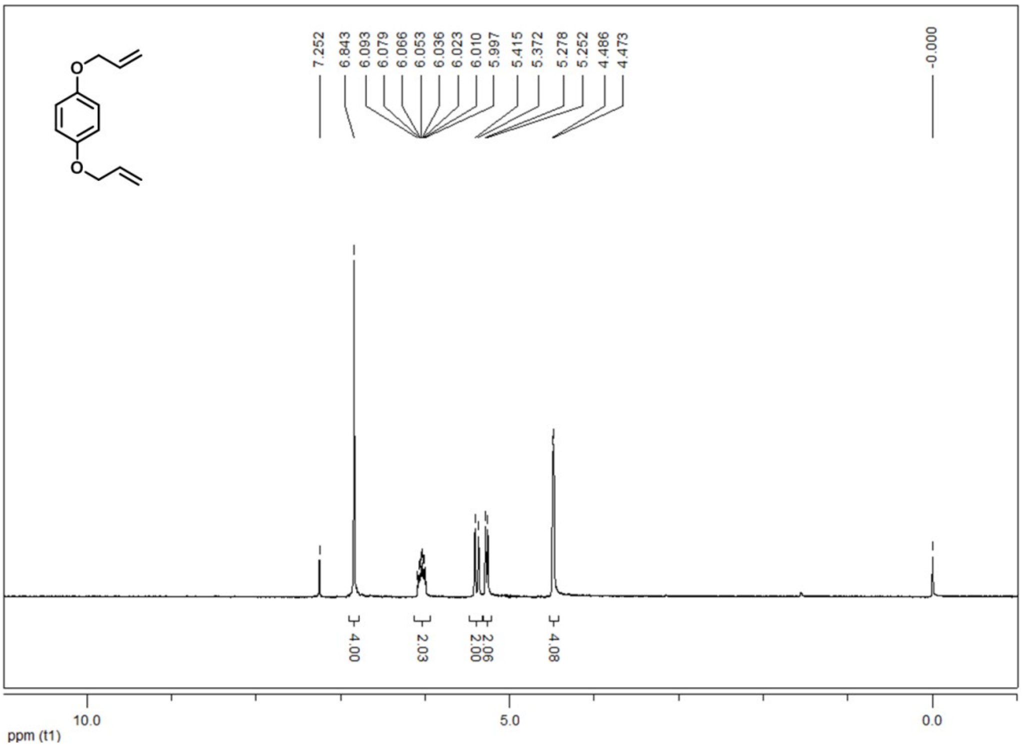

Figure S1. ^{1}H NMR spectrum ($CDCl_3$, 400 MHz, 298 K) of compound **1**.

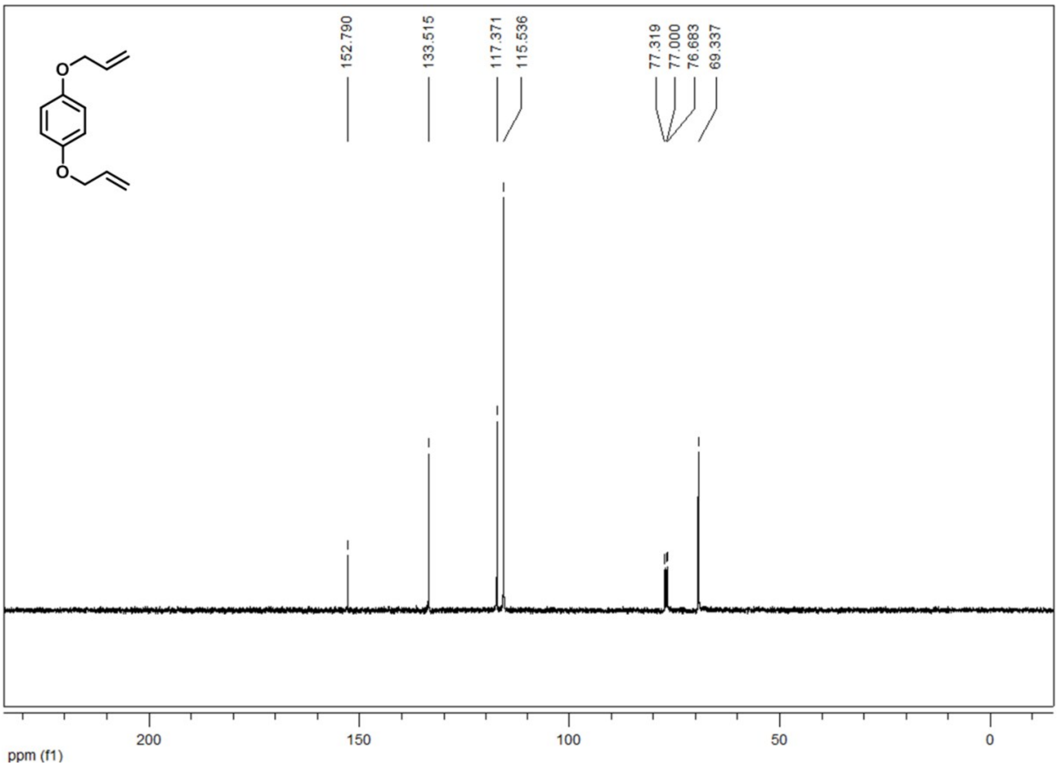

Figure S2. ^{13}C NMR spectrum ($CDCl_3$, 100 MHz, 298 K) of compound **1**.

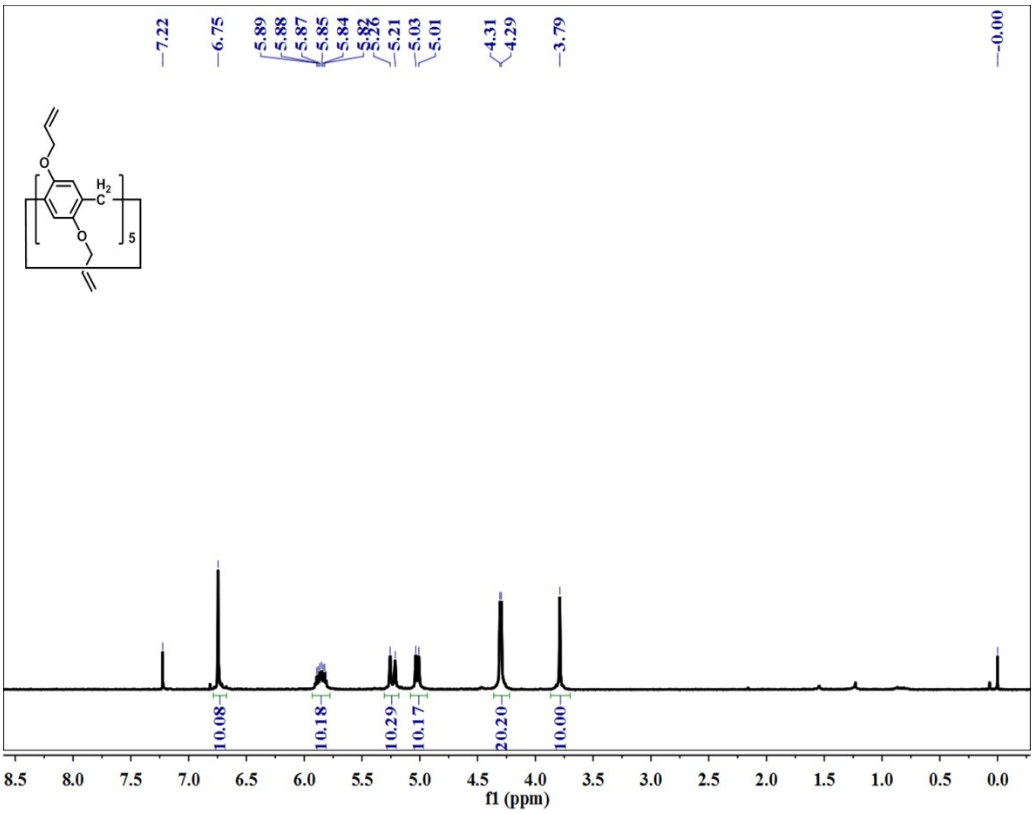

Figure S3. ^{1}H NMR spectrum ($CDCl_3$, 400 MHz, 298 K) of compound **2**.

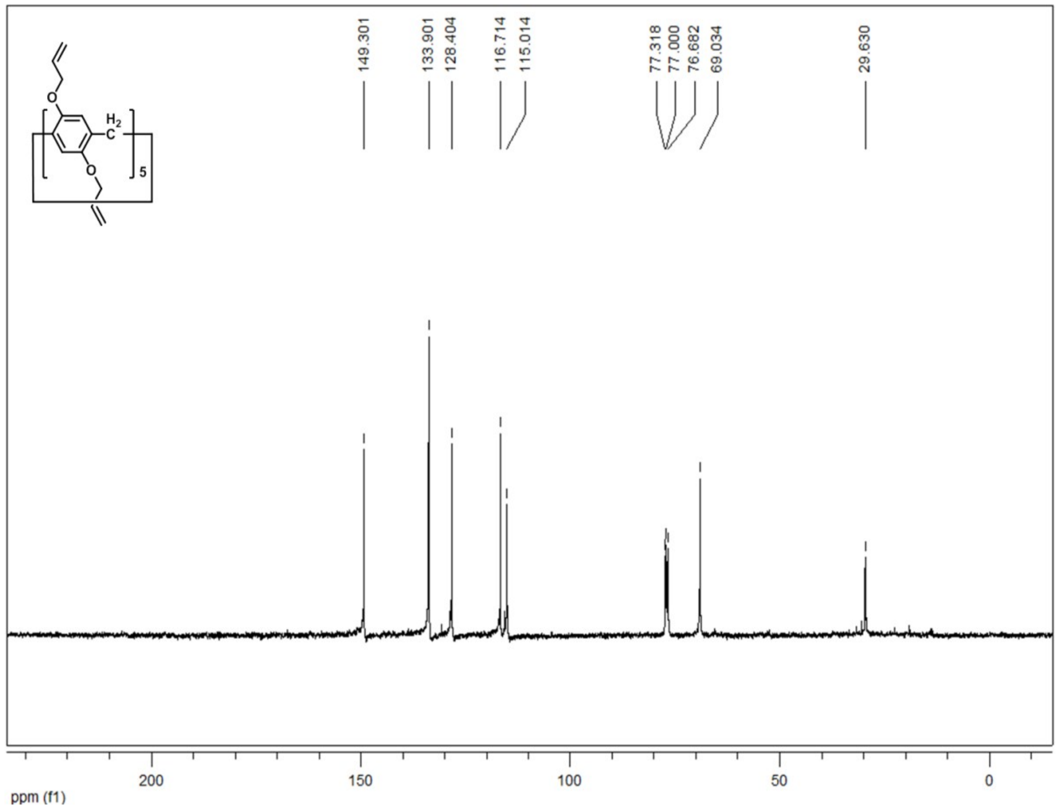

Figure S4. ^{13}C NMR spectrum ($CDCl_3$, 100 MHz, 298 K) of compound **2**.

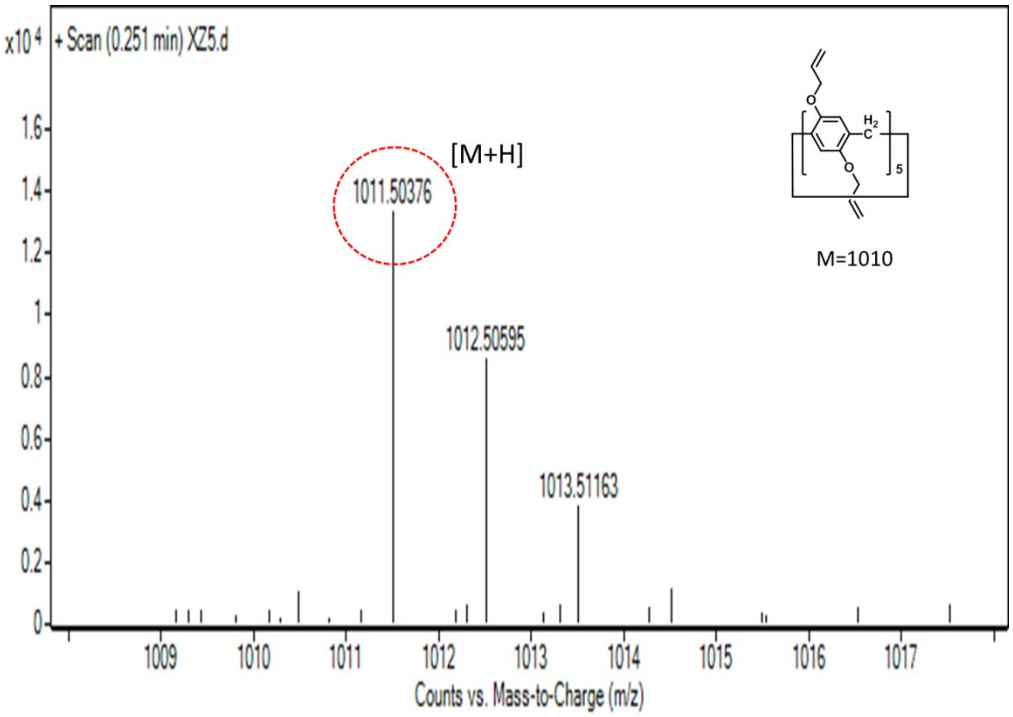

Figure S5. ESI-MS spectrum of compound **2**.

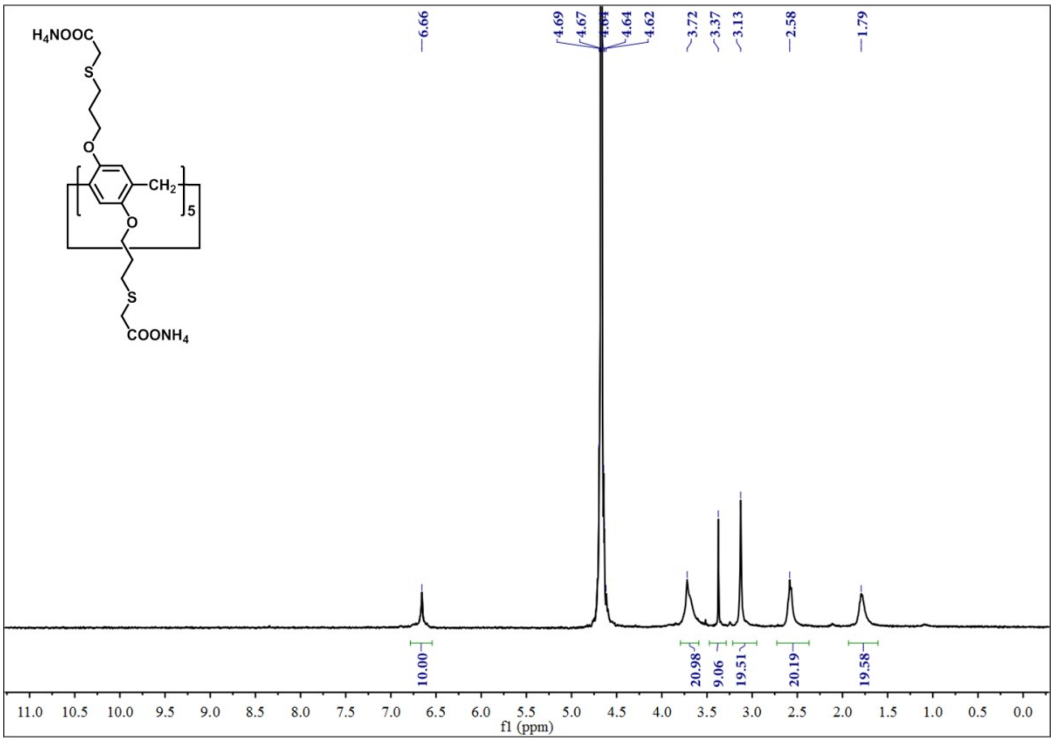

Figure S6. ^{1}H NMR spectra (D_2O, 400 MHz, 298 K) of mercaptoacetic acid-pillar[5]arene.

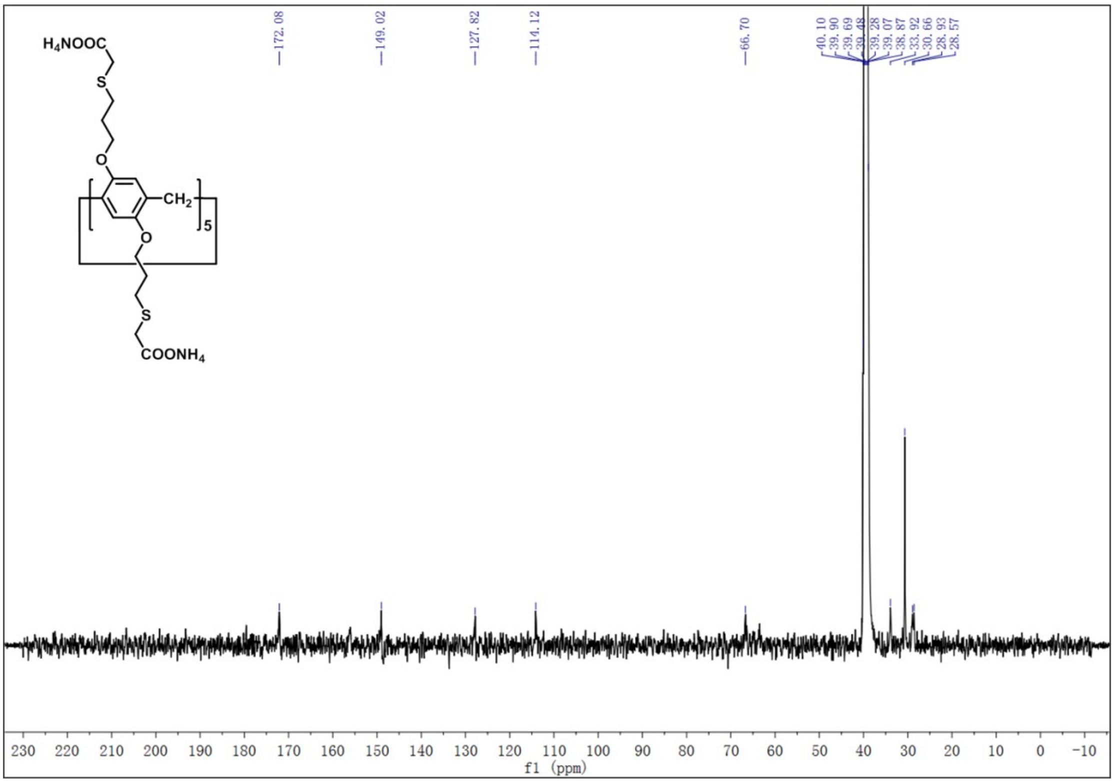

Figure S7. ^{13}C NMR spectra (DMSO-d_6, 100 MHz, 298 K) of mercaptoacetic acid-pillar[5]arene.

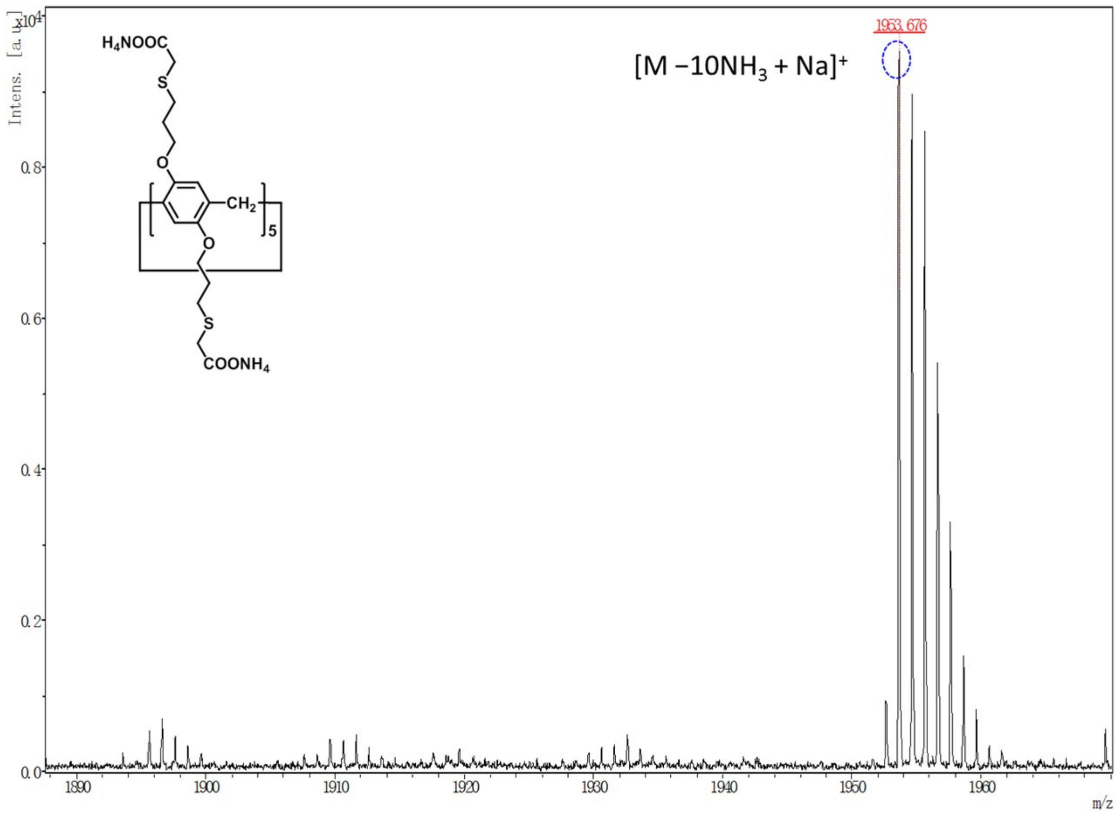

Figure S8. MALDI-TOF of mercaptoacetic acid-pillar[5]arene.

4. *^{1}H NMR for the host-guest interaction.*

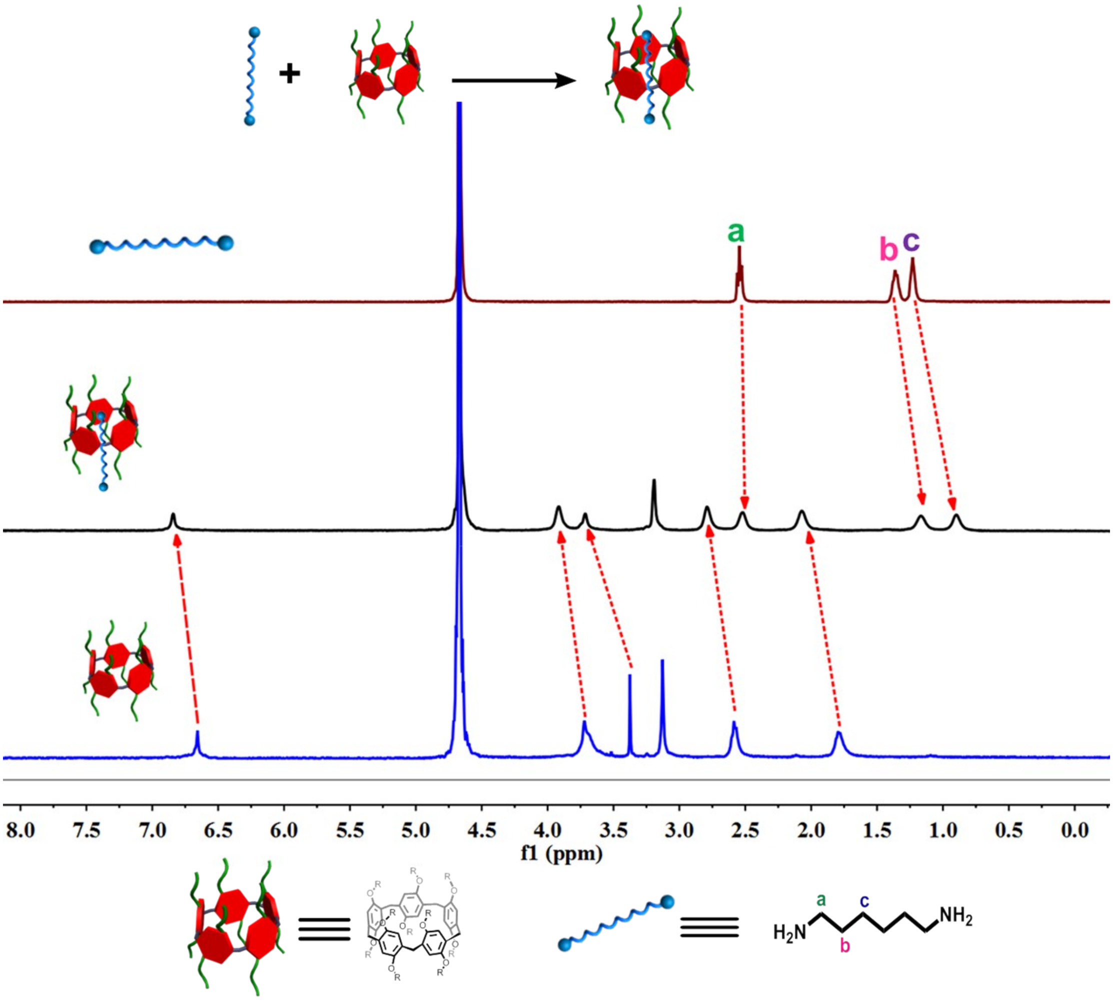

Figure S9. ^{1}H NMR spectra (D_2O, 400 MHz, 298 K) for the interaction of mercaptoacetic acid - pillar[5]arene and HDA. It shows that MAP5 and HDA can form a host-gust complex by the inclusion interaction.

Determination of the association constants.

To determine the stoichiometry and association constant (K_a) between MAP5 (host) and HDA (guest), ^{1}H NMR titrations were done with solutions which had a constant concentration of MAP5 (6.0 mM) and varying concentrations of HDA. By a mole ratio plot, a 1:1 stoichiometry was obtained, which indicated that MAP5 and HDA formed a 1:1 complex. Using the nonlinear curve-fitting method, the association constant was obtained for each host-guest combination from the following equation : [S3]

$$\Delta\delta = (\Delta\delta_\infty/[H]_0)\ (0.5[G]_0 + 0.5([H]_0+1/K_a) - (0.5\ ([G]_0^2+(2[G]_0(1/Ka - [H]_0)) + (1/Ka + [H]_0)^2)^{0.5}))$$

Where $\Delta\delta$ is the chemical shift change of H_1 of benzene in MAP5 at $[G]_0$, $\Delta\delta_\infty$ is the chemical shift change of H_1 when the host is completely complexed, $[H]_0$ is the fixed initial concentration of the host MAP5, and $[G]_0$ is the initial concentrations of guest HDA.

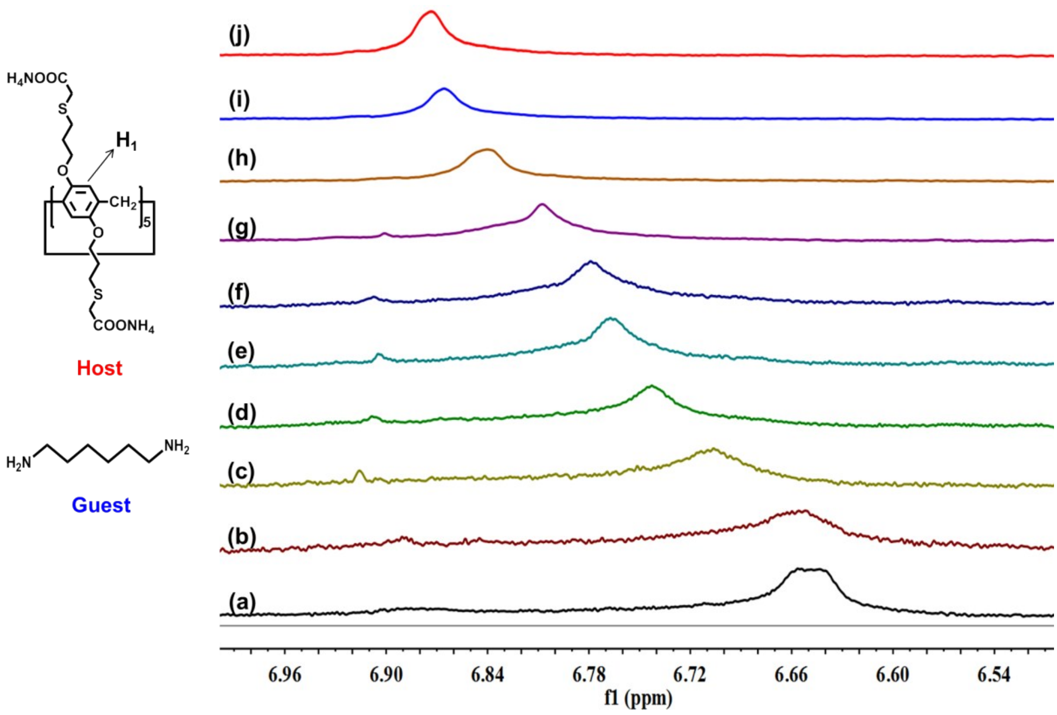

Figure S10. Partial ^{1}H NMR spectra (D_2O, 298 K, 400 MHz) of MAP5 at a concentration of 6 mM upon addition of different concentrations of 1: (a) 0.00 mM, (b) 0.498 mM, (c) 1.002 mM, (d) 1.98 mM, (e) 3.0 mM, (f) 4.002 mM, (g) 4.998 mM, (h) 6.0 mM, (i) 7.32 mM (j) 12.498 mM.

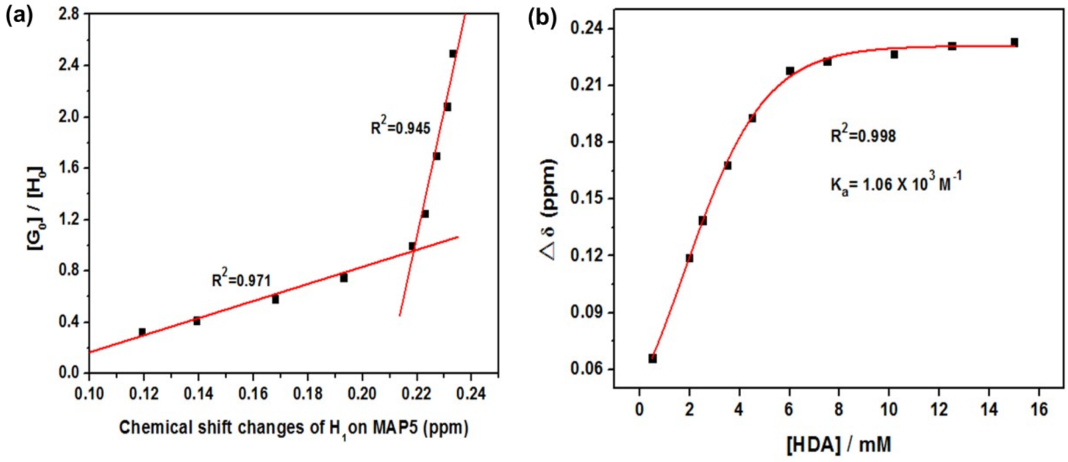

Figure S11. (a) Mole ratio plot for the complexation between MAP5 and HDA, indicating a 1:1 stoichiometry. (b) The non-linear curve-fitting (NMR titrations) for the complexation of MAP5 (6.0 mM) with different concentration of HDA. The association constant (K_a) of MAP5 and HDA was calculated to be 1.06×10^3 M^{-1}.

5. *Gaussian calculation for the interaction of MAP5 and HDA.*

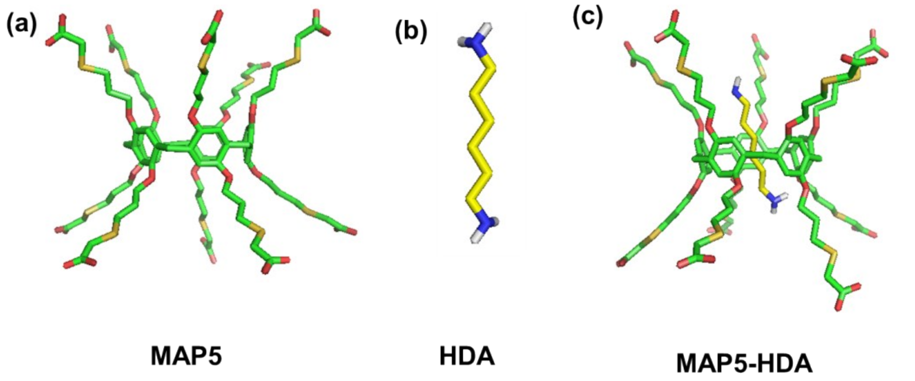

Figure S12. The optimized structure of (a) MAP5 (b) HDA and (c) the inclusion complex of MAP5 and HDA.

Table S1. Energy change of MAP5 and HDA by the computational calculations

Item	Energy（kJ / mol）
Host (MAP5)	-2.50×10^7
Guest (HDA)	-9.13×10^5
Complex (Host-Guest)	-2.59×10^7
△E	-30.456

△E = E(Complex)-E(MAP5)-E(HDA)

6. *UV-vis characteristic for the selective of mercaptoacetic acid-pillar[5]arene to metal ions.*

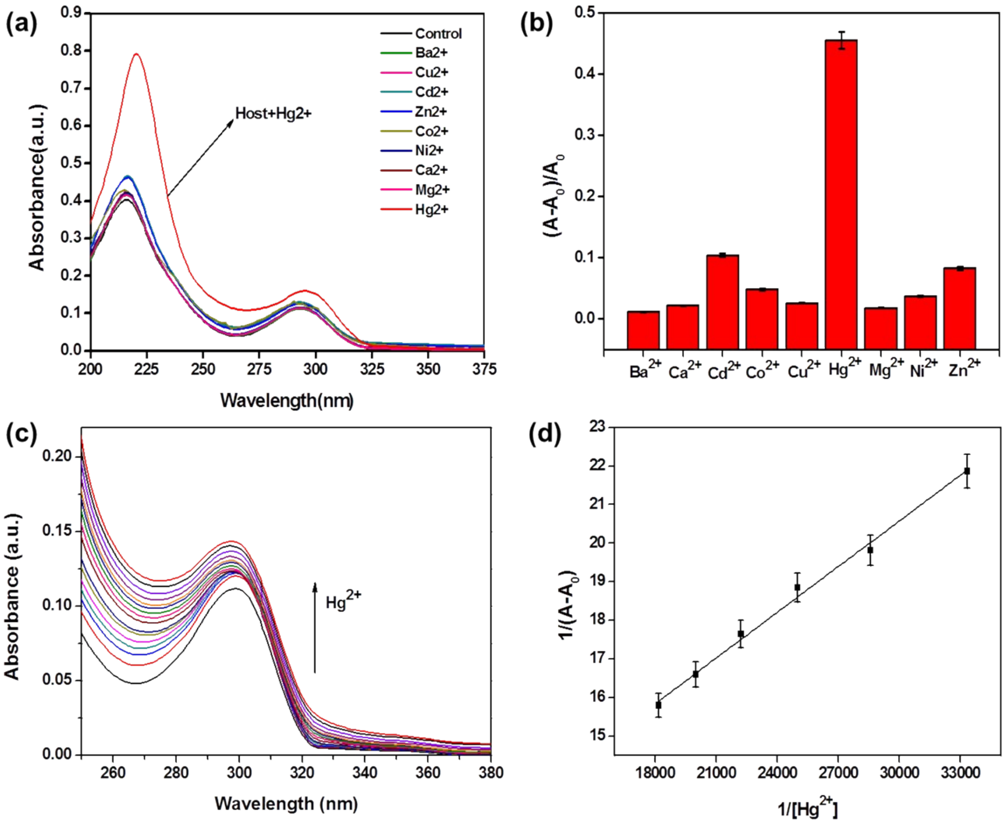

Figure S13. (a) UV-vis absorption of mercaptoacetic acid-pillar[5]arene (1×10^{-5} M) upon addition of different metal ions. (b) Histogram showing absorbance change of 294nm. A_0 is the absorbance of the mercaptoacetic acid-pillar[5]arene, A is the absorbance of after adding metal ions. (c) UV-vis spectra titration of MAP5 (1×10^{-5} M) with various equivalents of Hg^{2+} in H_2O. (0, 0.2, 0.4, 0.6, 0.8, 1, 1.5, 2, 2.5, 3, 3.5, 4, 5, 6, 8 equiv) (d) Linear curve-fitting of the uv-vis changes for the complexation of MAP5 and Hg^{2+}. The association constant (K_a) of MAP5 and Hg^{2+} was calculated to be 2.24×10^4 M^{-1} by Benesi-Hilderbrand equation. The results show that MAP5 have good selectivity for Hg^{2+}.

7. *Contact angles measurement.*

Contact angles were measured using an OCA20 (DataPhysics, Germany) contact-angle system at ambient temperature and saturated humidity. The original PET membrane for contact angle measurement was treated with etching solution (9 M NaOH) for 1h. The membrane was then taken out from the etching solution and treated with a stopping solution (1 M KCl + 1 M HCOOH) for 30 min. After that, the membrane was immerged in deionized water overnight. The process of modification is the same with the inner wall of nanochannel. Before the contact angle test, the sample was blown dry with N_2. In each measurement, an about 1 μL droplet of water was dispensed onto the surface of PET membrane. The average contact angel value was obtained at five different positions of the same membrane.

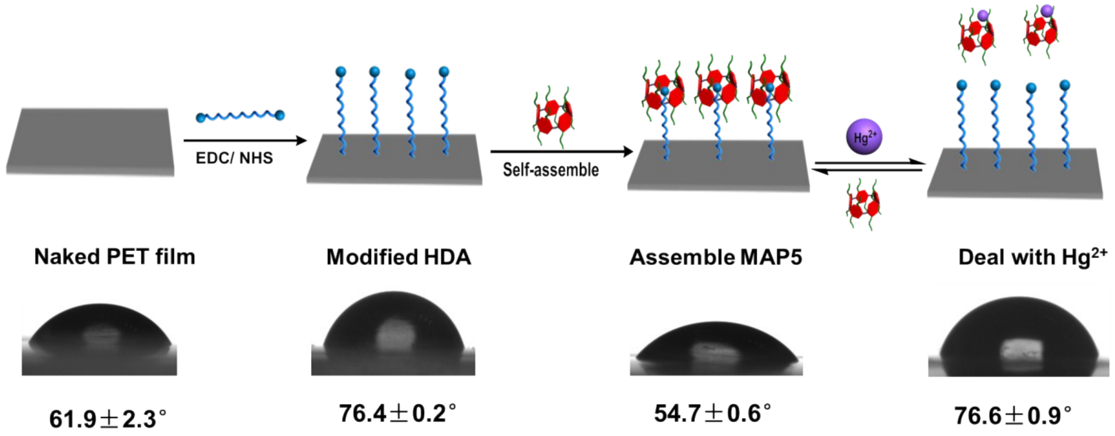

Figure S14. Water-drop profiles on the PET membrane surface for the modification of HDA and self-assemble with mercaptoacetic acid -pillar[5]arene. The result shows that MAP5 can be well immobilization to the nanochannel surface by self-assembly.

8. *XPS.*

X-ray photoelectron spectra (XPS) data were obtained with an ESCALab220i-XL electron spectrometer from VG Scientific using 300 W Al Kα radiation. All peaks were referenced to C1s (CHx) at 284.8 eV in the deconvoluted high resolution C1s spectra. The analysis software was used as provided by the instrument's manufacturer. The nitrogen element existed in the modified PET film indicates that 1,6-hexanediamine (HDA) was modified on the membrane successfully. And then the sulfur element existed in the modified PET film indicates that mercaptoacetic acid-pillar[5]arene (MAP5) was binding on the membrane.

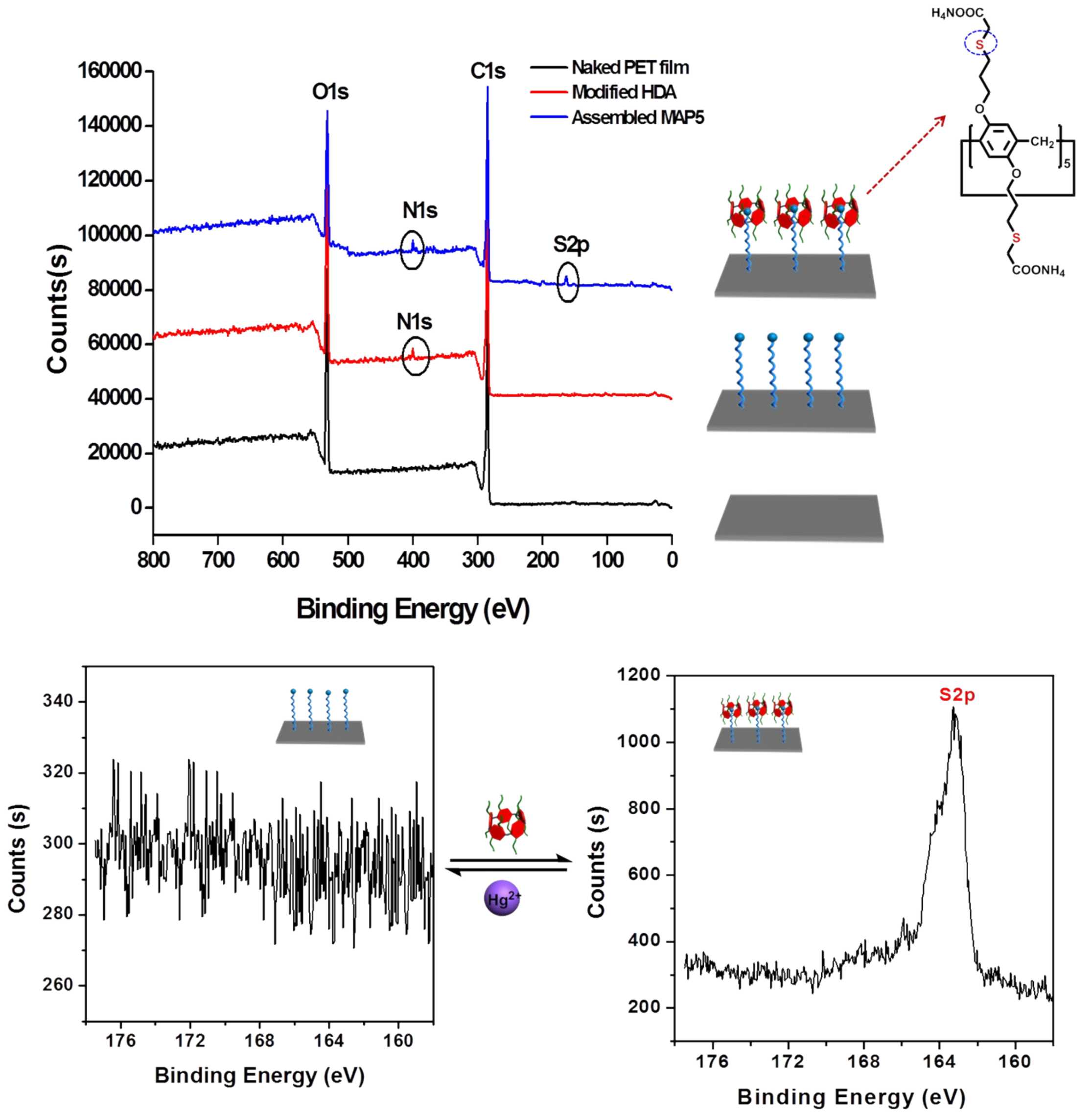

Figure S15. XPS characteristic for the modification and assembling process and details for the elements peaks. These results showed that MAP5 can be well assembled to the surface and regulated by Hg^{2+}.

Table S2 The XPS data of the PET film before modification

Name	Start BE	Peak BE	End BE	Height CPS	FWHM eV	Area(P) CPS.eV	Atomic %
C1s	295.37	284.83	280.42	26576.54	1.18	48973.4	79.06
O1s	538	532.86	526.87	12746.53	2.23	30951.01	18.88

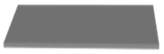

Table S3 The XPS data of the PET film after HDA modification

Name	Start BE	Peak BE	End BE	Height CPS	FWHM eV	Area(P) CPS.eV	Atomic %
C1s	293.82	284.83	281.42	23387.85	1.25	44473.38	72.22
O1s	536.51	531.89	527.07	14081.68	1.82	38929.4	23.88
**N1s**	**407.27**	**399.62**	**395.02**	**1014.34**	**1.37**	**2043.06**	**2.05**

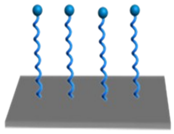

Table S4 The XPS data of the HDA-modified PET film assembled with mercaptoacetic acid - pillar[5]arene

Name	Start BE	Peak BE	End BE	Height CPS	FWHM eV	Area(P) CPS.eV	Atomic %
C1s	290.72	284.73	280.82	18331.51	1.37	35774.18	69.43
O1s	536.33	531.66	526.52	10145.03	2.8	28374.2	20.8
**N1s**	**404.02**	**399.62**	**395.57**	**903.76**	**1.38**	**1658.44**	**1.99**
**S2p**	**173.22**	**163.23**	**160.95**	**767.05**	**1.72**	**1814.24**	**1.81**

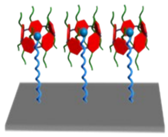

9. *Nanochannel fabrication*

The single conical nanochannel was prepared in a PET polymer film using the well-known ion track etching technique. Before etching process, each side of the PET membranes were exposed in UV light (365 nm) for 1hour. In order to obtain the conical nanochannel, etching was performed only from one side, the other side of the cell contains a solution that is able to neutralize the etchant as soon as the pore opens, thus slowing down the further etching process. The PET membrane was embedded between the two chambers of a conductivity cell at 30 ℃, one chamber was filled with etching solution (9 M NaOH), the other chamber was filled with stopping solution (1 M KCl + 1 M HCOOH). Then a voltage of 1 V was applied across the membrane. The etching process was stopped at a desired current value corresponding to a certain tip diameter. The membrane was immerged in MilliQ water (18.2 MΩ) to remove residual salts. The diameter of large opening of conical nanochannel which was called base (D) was determined by scanning electron microscopy (SEM). The diameter of the small opening which was called tip (dtip) was estimated by the following relation:

$$d_{tip} = \frac{4LI}{\pi k(c)UD}$$

L is the length of the pore, which could be approximated to the thickness of the membrane after chemical etching; *I* is the measured ion current; *U* is the applied voltage; d_{tip} and D is the tip diameter and the base diameter respectively; k(c) is the specific conductivity of the electrolyte. For 1 M KCl solution at 25 ^{o}C, k(c) is 0.11173 Ω^{-1} cm^{-1}. In this work, the base diameter is about 560 nm and the tip diameter is about 20 nm.

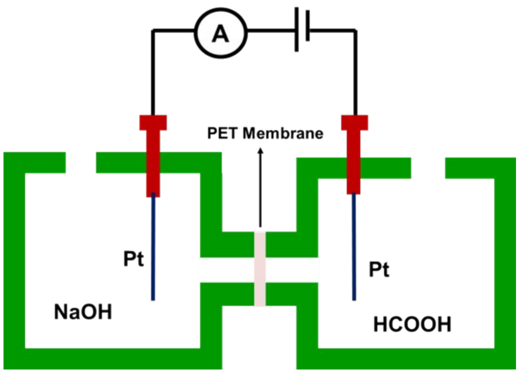

Figure S16. Schematic image for etching conical nanochannel in a conductivity cell.

10. *SEM Characterization*

The diameter of the base was estimated from the multitrack membrane by field-emission scanning electron microscopy (FESEM) which was etched under the same conditions as the single-channel sample. In this work, before modification the base diameter was about 560 nm and tip diameter was estimated by the above relation, tip was about 20 nm. In addition, the cross-section of the nanochannel shows the conical shape indicated that the conical nanochannel successfully prepared.

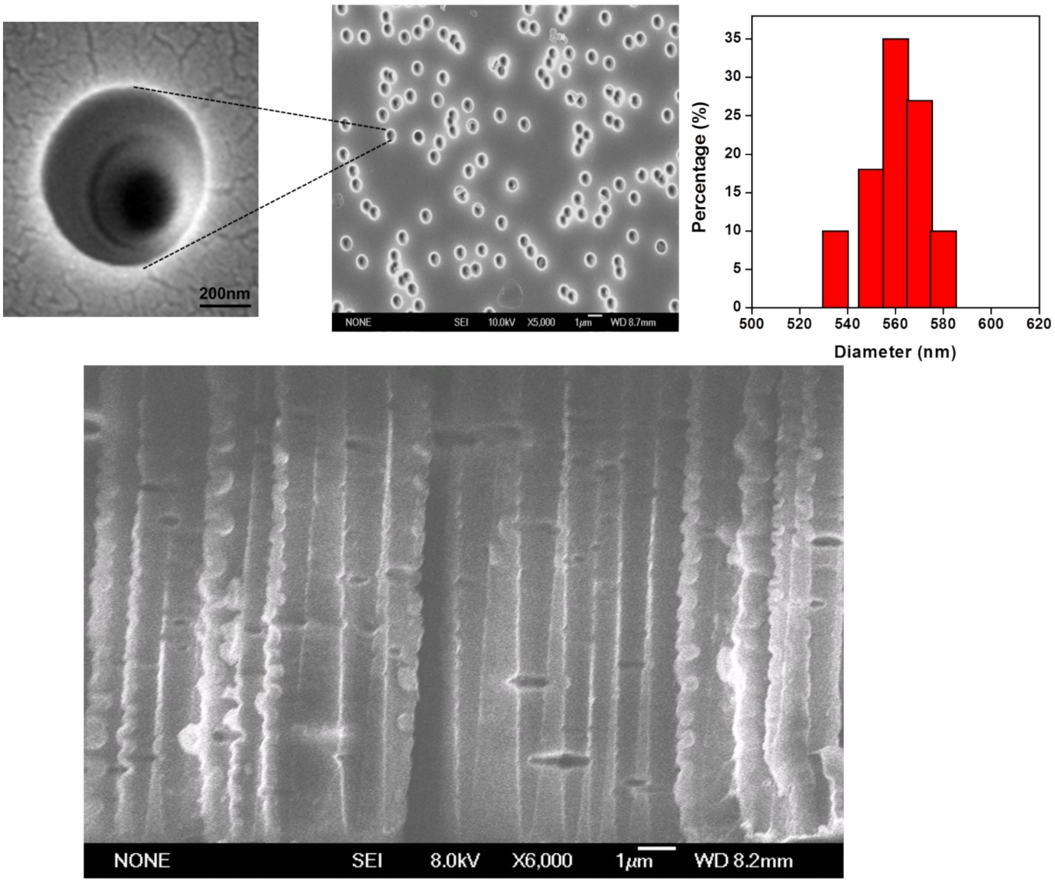

Figure S17. SEM image of the base side and cross-section of the conical nanochannel in PET porous membrane channels (10^7channels cm^{-2})

11. *Modification process*

As a result of chemical etching, carboxyl groups are generated on the nanochannel surface. These can be activated with EDC/NHSS, forming an amine-reactive ester intermediate. Then these reactive esters were further condensed with HDA through the formation of covalent bonds. In this paper NHSS ester was formed by soaking PET film in an aqueous solution of 15 mg EDC and 3 mg NHSS for 1 hour. After that washing this film with distilled water and treated it with 1 mM HDA solution overnight. Then, the mercaptoacetic acid-pillar[5]arene (MAP5) were attached to the HDA-channel by self-assembling. Finally, the modified film was washed three times with distilled water.

12. *Ion currents measurement*

Ion currents were measured by a Keithley 6487 picoammeter (Keithley Instruments, Cleveland, OH). Ag/AgCl electrodes were used to apply a transmembrane potential across the film. The film was mounted between the two halves of the conductance cell. Both halves of the cell were filled with a 0.1 M KCl, pH 6.86. In order to record the I–V curves, a scanning triangle voltage signal from -2V to +2V with a 40s period was selected. Each test was repeated 5 times to obtain the average current value at different voltage.

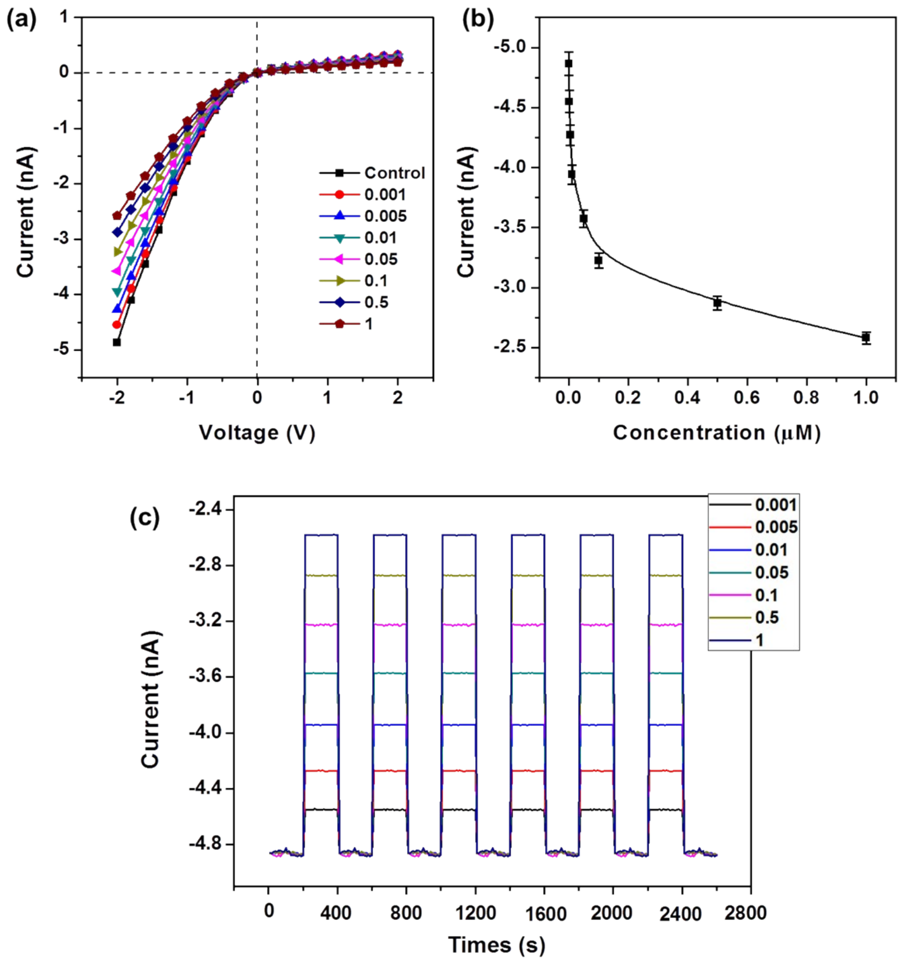

Figure S18. (a) I–V characteristics of MAP5-assembled nanochannel upon exposure to different concentrations of Hg^{2+} (1 nM to 1 μM). (b) Current–concentration properties of the MAP5 assembled channels in the presence of Hg^{2+}. (c) The current variation of ion nanochannels at -2.0 V in presence of different concentration of Hg^{2+}. The results show that with concentration increase, the K^+ current gradually decrease.

13. ***Laser scanning confocal microscopy***

In order to directly characterize the MAP5 assembled in the conical nanochannel, the host MAP5 was derived and the rhodamine B amine (RhB-NH_2) severed as the fluorophore was introduced. Briefly, the MAP5-RhB was synthesis as follows: 5 mL of MAP5 (1 mM) in water activated by 30mg EDC and 6 mg NHSS, then 5 mL of RhB-NH_2 (10 mM) in the buffer solution of PBS at pH = 5.5 was added in flask. Subsequently, the mixture reacted for 10 hours at room temperature. After purified, the MAP5-RhB assembled to the porous PET film according to the same method in the single nanochannel. And then the MAP5-RhB assemble porous PET film was immersed in 10^{-3} M Hg^{2+} solution for 1 hour. Before the measurement, the film was washed by distilled water. Fluorescence microscopic images were acquired by using a Zeiss confocal laser scanning unit mounted on a LSM710 fixed-stage upright microscope.

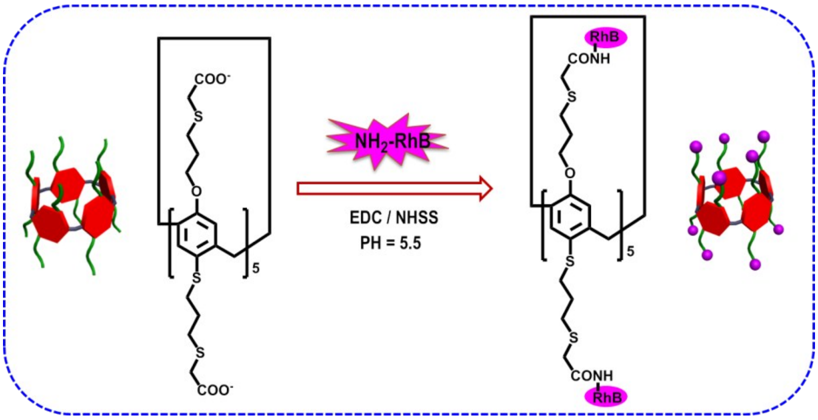

Figure S19. Prepare for the fluorescent derivative MAP5-RhB.

14. *References*

S1. Wang, Z. M.; Shen, M. *J. Org. Chem.***1998**, *63*, 1414-1418.

S2. Liu, L.; Cao, D.; Jin, Y.; Tao, H.; Kou, Y.; Meier, H. *Org. Biomol. Chem*. **2011**, *9*, 7007-7010.

S3. Li, C.; Zhao, L.; Li, J.; Ding, X.; Chen, S.; Zhang, Q.; Yu, Y.; Jia, X. *Chem. Commun.*, **2010**, *46*, 9016–9018.
